# Factors Associated with Timing of Initiation of Antiretroviral Therapy among HIV-1 Infected Adults in the Niger Delta Region of Nigeria

**DOI:** 10.1371/journal.pone.0125665

**Published:** 2015-05-01

**Authors:** Dimie Ogoina, Finomo Finomo, Tubonye Harry, Otonyo Inatimi, Ikenna Ebuenyi, Wolo-wolo Tariladei, Abimbola Anne Afolayan

**Affiliations:** 1 Department of Internal Medicine, Niger Delta University Teaching Hospital, Okolobiri, Bayelsa state, Nigeria; 2 Department of Medicine, Federal Medical Centre, Yenagoa, Bayelsa State, Nigeria; University of Texas Health Science Center San Antonio Texas, UNITED STATES

## Abstract

**Introduction:**

Based on growing evidence mainly from countries outside Sub-Saharan Africa, the World Health Organisation (WHO) now recommends initiation of antiretroviral therapy (ART) in HIV-infected individuals in developing countries when CD4 cell count (CD4+) is ≤ 500cells/ul. Nigeria accounts for about 14% of the estimated HIV/AIDS burden in Sub-Saharan Africa. We evaluated the factors associated with timing of initiation of ART among treatment-ineligible HIV-infected adults from Nigeria.

**Methods:**

We retrospectively reviewed the hospital records of ART ineligible HIV-infected adults who enrolled into HIV care between January 2008 and December 2012 at two major tertiary hospitals in Bayelsa State, South-South Nigeria. Demographic, clinical and laboratories data were obtained at presentation, at each subsequent visit at 6 monthly intervals and at time of initiation of ART. Cox proportional regression and Kaplan-Meier survival analysis were used to evaluate independent predictors of time to initiation of ART.

**Results:**

Amongst the 280 study participants, 70.6% were females, 62.6% had CD4+ ≥500cells/ul, 48.4% had WHO HIV Stage 1 disease and 34.3% were lost to follow up. In a cohort of 180 participants followed up for ≥3months, participants with CD4+ of 351-500cells/ul and stage 2 disease were more likely to start ART earlier than those with CD4+ > 500cells/ul (Hazard ratio [HR]-1.7, 95% confidence interval [CI] of 1.0-2.9) and stage 1 disease (HR-2.3 (95% CI-1.3-4.2) respectively. HIV-infected adults with faster CD4+ decay required earlier ART initiation, especially in the first year of follow up.

**Conclusion:**

ART-ineligible HIV-infected adults on follow up in South-South Nigeria are more likely to require earlier initiation of ART if they have stage 2 HIV disease or CD4+ ≤500cells/ul at presentation. Our findings suggest faster progression of HIV-disease in these groups of individuals and corroborate the growing evidence in support for earlier initiation of ART.

## Introduction

The optimal time to start antiretroviral therapy (ART) in HIV-infected individuals remains a subject of controversy and debate. In its most recent updated adult HIV treatment guidelines for resource limited countries, the world health organisation (WHO) recommends initiation of ART in all individuals with CD4+ cell count of 500cells/ul or less, irrespective of the clinical stage of the disease [1]. This WHO review was necessitated by studies which showed that initiating ART at a CD4+ cell count > 350cells/ul compared with treatment at a CD4+ cell count ≤ 350cells/ul reduced the risk of progression to AIDS and/or death[2], reduced the risk of development of tuberculosis (TB)[3][4] and non-AIDS-defining illness[2], as well as increased the likelihood of immune recovery[2][5]. Furthermore, early initiation of ART has been shown to substantially reduce sexual transmission of HIV in serodiscordant couples[4].

Most of the studies necessitating this review were conducted in the developed world, with a dearth of studies from developing countries in Sub-Saharan Africa[6][7], a region that accounts for more than 60% of the world’s HIV/AIDS burden.

With about 3.2 million people living with HIV/AIDS, Nigeria accounts for about 14% of the estimated HIV/AIDS burden in Sub-Saharan Africa and 10% of global burden[8][9]. There are still substantial gaps in access to ART in Nigeria as out of about 1.5million HIV-infected individuals in need of highly active antiretroviral therapy (HAART) only approximately 360, 000 are currently receiving ART[9]. In order to ascertain the categories of ART ineligible individuals at risks of earlier initiation of ART and to provide data on possible clinical benefits of earlier initiation of ART in the Nigeria HIV-infected adult population, we determined the factors associated with timing of ART initiation among HIV-infected adults who were ineligible for ART at the time of enrolment into HIV-care in two tertiary hospitals in Nigeria. To our knowledge this is the first of such studies from Nigeria.

## Materials and Methods

### Study design and setting

We retrospectively reviewed the hospital records of ART ineligible HIV-infected adults who enrolled into HIV care between January 2008 and December 2012. Ineligibility for ART was defined as having a CD4+ cell count > 350cells/ul and WHO HIV stage 1 and 2 based on Nigerian National guidelines. [10]

The study was conducted in Federal Medical Centre, Yenagoa and Niger Delta University Teaching Hospital Okolobiri, which are two major tertiary hospitals situated in Bayelsa State, South-South Nigeria. Bayelsa state is located in the core of the Niger Delta region of Nigeria and has an HIV seroprevalence of 9.1%, the third highest in the country.

Both tertiary hospitals serve as major HIV/AIDS treatment and referral centres for the state and about four other states in the Niger Delta region of Nigeria.

### Inclusion and exclusion criteria

We included all HIV-infected adults who at baseline were ineligible for ART initiation and excluded all pregnant women, or women who were started on ART on account of pregnancy.

### Study participant monitoring and follow-up

Following HIV testing, all HIV-infected clients are usually offered HIV post-test counselling by a trained counsellor, clinically evaluated by a physician and then sent to the laboratory for baseline investigations, including CD4+ cell count. All our study participants were HIV-1 infected confirmed by enzyme linked immunosorbent assay (ELISA). Western blot and HIV viral loads are not routinely done in both hospitals due to resource constraints.

After baseline investigations, clients who are ineligible for ART are asked to return for regular clinical assessments and CD4+ cell counts every three to six months. As soon as commencement of ART is indicated according to National treatment guidelines[10], the HIV-infected clients are evaluated and initiated on appropriate ART drug combinations.

### Data collection

Data was collected from case records of all study participants fulfilling inclusion criteria through a structured questionnaire completed by four trained research assistants. Relevant demographic, clinical and laboratory data at baseline and at every three to six month visits were documented. Clinical diagnosis and the WHO HIV stage according to established guidelines[1] were also documented at baseline and at time of initiation of ART. Laboratory results obtained at baseline included creatinine clearance (CrCl:calculated by Cockcroft Gault formula), CD4+ cell counts (by flow cytometry), packed cell volume (PCV) and fasting blood sugar (FBS). The CD4+ cell counts at three to six monthly intervals were documented when available. Although, majority of study participants were receiving cotrimoxazole (Septrin) prophylaxis, the regularity of prophylaxis could not be assessed due to inconsistent documentation in case records. Probable or confirmed cases of death during the study period were not analysed due to paucity of required data in study participant records.

### Follow up Outcome variables

There were four possible outcomes on follow-up of participants, including those who started ART, those yet to start ART as at December 2012, those lost to follow up (LTFU), and those transferred. LTFU was defined as failure to return to care after three months from the last scheduled visit or throughout the duration of the study. The pre-ART and ART-registers were thoroughly scrutinized during the study period to identify participants who might have returned to care after a period of absence.

Study participants qualified to start ART when they developed WHO HIV stage 3 or 4 disease or when the CD4+ cell count drop to 350cells/ul or below.

### Statistics

Demographic and clinical data were summarised using percentages and median (with inter-quartile ranges-IQR). Differences in baseline demographic and laboratory variables according to ART status (i.e. started ART and yet to start ART) in study participants followed up for at least three months were compared by Chi square and Mann-Whitney test as appropriate. For all statistical analyses, study participants who were yet to start ART at the time of lost to follow up or transfer but followed up for at least three months were also classified as ‘yet to start ART’. We compared six- monthly median CD4+ cell count according to ART status by Mann Whitney test. Independent predictors of starting ART were determined by an unconditional binary logistic regression model including possible predictor variables at baseline.

Time to start of ART was assessed by Kaplan–Meier analysis using the log-rank test to compare CD4+ cell count group and WHO stages 1& 2. Independent predictors of time to initiation of HAART in study participants followed up for at least 3months were determined by a Cox proportional regression analysis including possible predictor variables from univariate analysis such as WHO HIV stage (Stage 1 & Stage 2), CD4+ cell count group (351–500 & >500cells/ul), creatinine clearance (<90mls/min & ≥90mls/min), age and sex. Only baseline variables were used for the regression analysis. Statistical package for social sciences (SPSS) version 20 was used for all analyses. P<0.05 was statistically significant.

### Ethics

Ethical approval was sought for this study and obtained from the Federal Medical Centre, Yenagoa and Niger Delta University Teaching Hospital Okolobiri Research and Ethics committees. To ensure confidentiality of participant’s information, we anonymized and de-identified all study data of our participants prior to analysis.

## Results

A total of 280 study participants, including 83males and 197 females with overall median age (IQR) of 31years (26, 38) were studied. Of the 280 study participants, 62.3% were ever married and 71.9% reported heterosexual sex as the most likely HIV risk factor.

### Clinical characteristics of study participants

The 280 study participants were followed up for a median period (IQR) of six months (1, 12), with minimum duration of follow up of 1month and maximum duration of 48months. According to WHO HIV staging, 136 (48.7%) of 280 study participants had WHO stage 1 HIV-disease while 144 (51.3%) had stage 2 HIV-disease.

The median PCV, CrCl, FBS and CD4+ cell count (and IQR) were 35% (32, 38), 85.8mls/min (70.3, 108.5), 4.7mmol/l (4.2, 5.4) and 554cells/ul (461,696) respectively.

With regard to CrCl, 40.9% of study participants had CrCl≥90mls/min while 59.1% had CrCl<90mls/min. With regard to CD4+ cell count groups, 37.5% of 280 study participants had CD4+ cell count of 351-500cells/ul while 62.5% had CD4+ cell counts >500cells/ul.

### Outcome of follow up

The number of study participants yet to start ART at 0, 3, 6, 12, 24, 36 and 48 months were 280, 186, 165, 94, 29, 10 and 1 respectively. Out of the 280 study participants, 94 (33.6%) started ART, 86 (30.7%) were yet to start as at December 2012, 96 (34.3%) were LTFU and 4 (1.4%) were transferred to other centres. The numbers and percentages of study participants according to outcome of follow up and duration of follow up are shown in Table 1. Of the 96 LTFU, the majority (80.2%) occurred after one month of follow up. LTFU was not significantly associated with any of the study variables (S1 Results).

**Table 1:** Distribution of study participants according to outcome and duration of follow up.

Months of follow up	Outcome of follow up				
	Started ART (n = 94) N (%)	Yet to start ART (n = 84) N (%)	LTFU (n = 96) N (%)	Transferred (n = 4) N (%)	Total (n = 280) N(%)
<3 months	2 (2.1)	10 (11.6)	77 (80.2)	3 (75)	92 (32.9)
3 months	5 (5.3)	2 (2.3)	1 (1.0)	0	8 (2.9)
4–6 months	20 (21.3)	14 (16.2)	3 (3.1)	0	37 (13.2)
7-12months	27 (28.7)	30 (34.9)	7 (7.3)	1 (25)	65 (23.2)
13-18months	20 (21.3)	9 (10.5)	6 (6.3)	0	35 (12.5)
19-24months	9 (9.6)	11 (12.8)	0	0	20 (7.1)
25-30months	7 (7.4)	3 (3.5)	1 (1.0)	0	11 (3.9)
31-36months	2 (2.1)	3 (3.5)	1 (1.0)	0	6 (2.1)
37-42months	1 (1.1)	2 (2.3)	0	0	3 (1.1)
43-48months	1 (1.1)	2 (2.3)	0	0	3 (1.1)

Table footnote: ART-antiretroviral therapy, LTFU-Lost to follow-up, n-number

### Review of study participants followed-up for 3months to 48months

Of the 280 study participants, 186 (66.3%) were followed up for at least three months, 90 (48.4%) started ART and 96 (51.6%) were yet to start ART by December 2012 or at time of LTFU/transfer. The differences in baseline demographic and clinical variables of this group of study participants according to ART status are shown in Table 2. Study participants who started ART are significantly more likely to have stage 2 HIV-disease, lower CD4+ cell count ranges (351-500cells/ul), and lower CrCl of less than 90mls/min (Table 2) than study participants who were yet to start ART. Study participants age, sex, FBS, and months of follow-up were not significantly related to ART status (p>0.05).

**Table 2:** Baseline Demographic and clinical variables of study participants in relation to ART status.

Variables	Started ART	Yet to start ART	Total	P value (differences in ART status)
Age in years (Median/IQR)	32 (28,39) n = 90	30 (26,38) n = 96	31 (27,39) n = 186	0.24
**Gender (N/%)**				0.56
Male	27 (54%)	23 (46%)	50 (26.9%)	
Female	63 (48.4%)	73 (53.7%)	136 (73.1%)	
**WHO HIV stage (N/%)**				0.015
Stage 1	35 (39.3%)	54 (60.7%)	89 (47.8%)	
Stage 2	55 (56.7%)	42 (43.3%)	97 (52.1%)	
**CD4 cell count (N/%)**				<0.0001
351-500cells/ul	48 (64.9%)	26 (35.1%)	74 (39.8%)	
>500 cells/ul	42 (37.3%)	70 (62.5%)	112 (60.2%)	
**CrCl (N/%)**				0.011
≥90mls/min	14 (29.8%)	33 (70.2%)	47 (37.6%)	
<90mls/min	41 (52.6%)	37 (47.4%)	78 (62.4%)	
Baseline CD4 cell count in cells/ul (Median/IQR) (n = 186)	491 (429,613)	600 (493,733)	544 (461, 682)	<0.0001
PCV (Median/IQR) (n = 153)	34 (31,37.7)	35 (32,37.9)	35 (32,37.9)	0.22
FBS(Median/IQR) (n = 118)	4.6 (3.8,5.3)	4.8 (4.3,5.4)	4.6 (4.2,5.3)	0.09
Follow up in months (n = 186)	12 (6,18)	12 (7,18)	12 (7,18)	0.9

Table footnote: ART-antiretroviral therapy, IQR-inter-quartile range, CrCl-creatinine clearance, n-number, PCV-packed cell volume, FBS-fasting blood sugar

### Clinical Presentation at Time of ART initiation

Out of the 90 study participants who started ART, 40 (44.4%) were asymptomatic (started ART on account of fall in CD4+ cell count alone), while 50 (55.6%) were symptomatic.

The clinical presentations of these symptomatic participants include pulmonary tuberculosis (n = 11), weight loss >10% of body weight (n = 12), chronic diarrhoea (n = 9), recurrent fever (n = 4) and pneumonia (n = 4). Two participants each had typhoid fever, upper respiratory tract infection and oral thrush. One participant each had vaginal discharge syndrome, sepsis, peripheral neuropathy, otitis media, and nodular prurigo, as well as disseminated tuberculosis and chronic hepatitis B infection.

Study participants with baseline CD4+ cell count of 351-500cells/ul were more likely to have symptomatic disease (i.e. WHO HIV stage 3 or 4) than those with baseline CD4+ cell count > 500cells/ul (Odds ratio-2.5, 95% CI-1.3–4.9, p = 0.007).

### HIV clinical staging on starting ART and progression to AIDS

Of the 90 study participants who started ART, 35 (39.3%) and 55 (56.7%) had stage 1 and 2 HIV diseases respectively at baseline. At the end of the study, 14 (15.6%), 45 (50%), 29 (32.2%) and 2 (2.2%) of the 90 participants had evidence of stage 1, 2, 3 and 4 HIV diseases.

### Independent predictors of starting ART

Following binary logistic regression analysis including age, sex, CD4+ cell count group, WHO HIV stage, and CrCl group as potential predictor variables, the CD4+ cell count group and the WHO HIV stage were the only independent predictors of starting ART. Study participants with WHO HIV Stage 2 were three times more likely to start ART than those who had stage 1 (Odds ratio [OR]-2.8, 95% Confidence Interval [CI]-1.25–6.31, p = 0.012). Study participants with CD4+ cell count range of 351-500cells/ul were also three times more likely to start ART than those with CD4+ cell counts of greater than 500cells/ul (OR-3.38, 95% CI-1.52–7.49, p = 0.003). Other variables including age, (OR-1.0, 95% CI-0.95–1.06), gender (male versus female; OR-1.84, 95% CI-0.69–4.89), and CrCl (≥ 90mls/min versus < 90mls/min versus; OR-0.47, 95% CI-0.19–1.10) were not independently associated with starting ART.

### Time to initiation of ART and associated predictor variables

Overall the median time from baseline to start ART (median ART initiation time) for the 186 study participants was 18months (CI-13.8–22.2). The predictors of time to ART initiation as determined by Cox proportional regression analyses are shown in Table 3. WHO HIV staging and CD4+ cell count range were the only variables independently associated with earlier initiation of ART. Study participants with WHO HIV Stage 2 were more likely to start ART earlier than those who had stage 1 (Hazard ration [HR]-2.3, 95% Confidence Interval [CI]-1.26–4.21, p = 0.007). Study participants with CD4+ cell count range of 351-500cells/ul were more likely to start ART earlier than those with CD4+ cell counts of greater than 500cells/ul (HR-1.7, 95% CI-1.01–2.98, p = 0.047).

**Table 3:** Predictors of time to ART initiation as determined by Cox proportional regression analysis.

Variable	Hazard ratio	95% Confidence interval	P value
Age	1.0	0.97–1.03	0.93
Gender			
Male	1.85	0.97–3.54	0.06
Female	1		
**WHO HIV staging**			
Stage 2	2.3	1.26–4.21	0.007
Stage 1	1		
**CD4 cell count (cells/ul)**			
351–500	1.73	1.01–2.98	0.047
>500	1		
**Creatinine clearance**			
CrCl<90mls/min	1.33	0.7–2.53	0.39
CrCl≥90mls/min	1		

Footnote: CrCl-creatinine clearance

**Fig 1:**
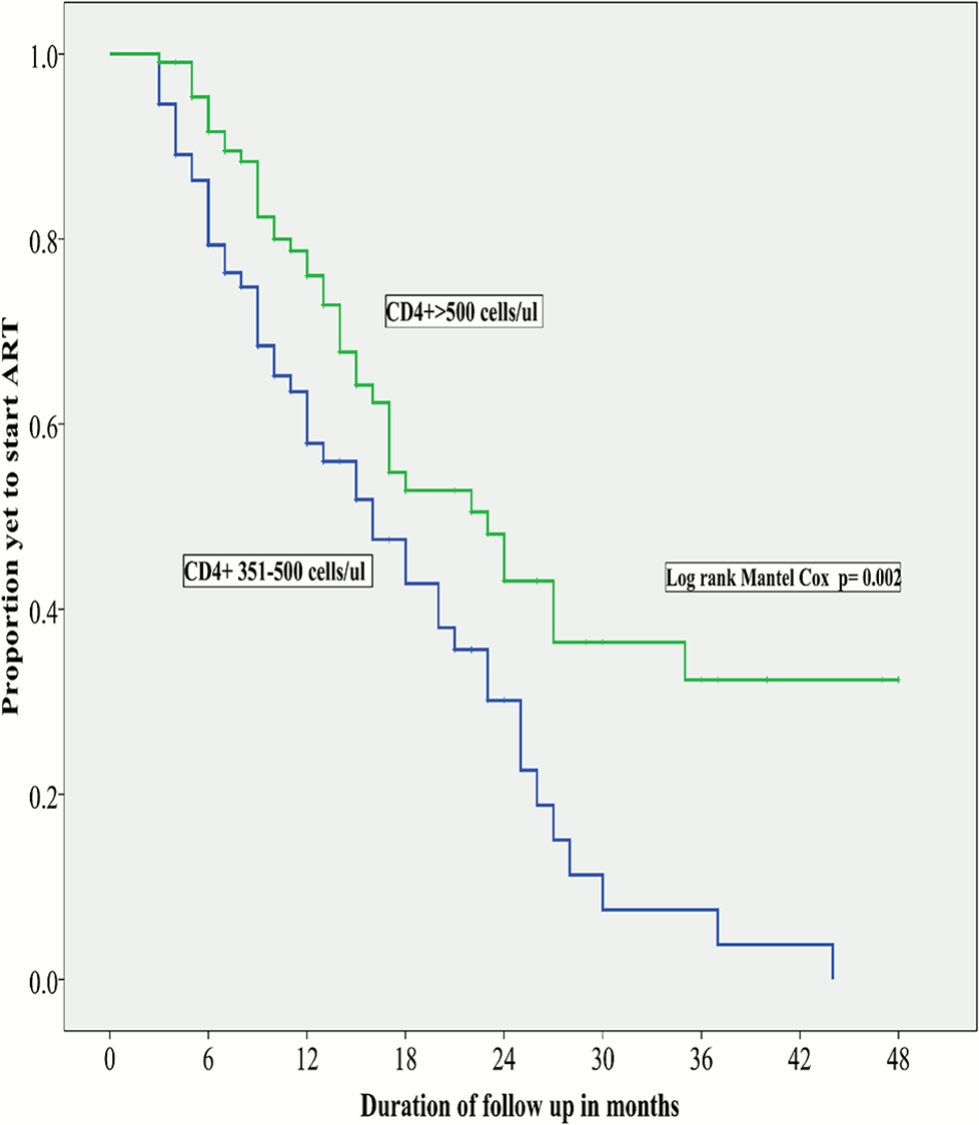
Kaplan-Meier curves of time to initiation of ART in relation to CD4 cell count group. The median time to ART initiation was significantly shorter in participants with CD4 cell count of 351-500cells/ul than those (16months) with CD4 count >500cells/ul (23months).

**Fig 2:**
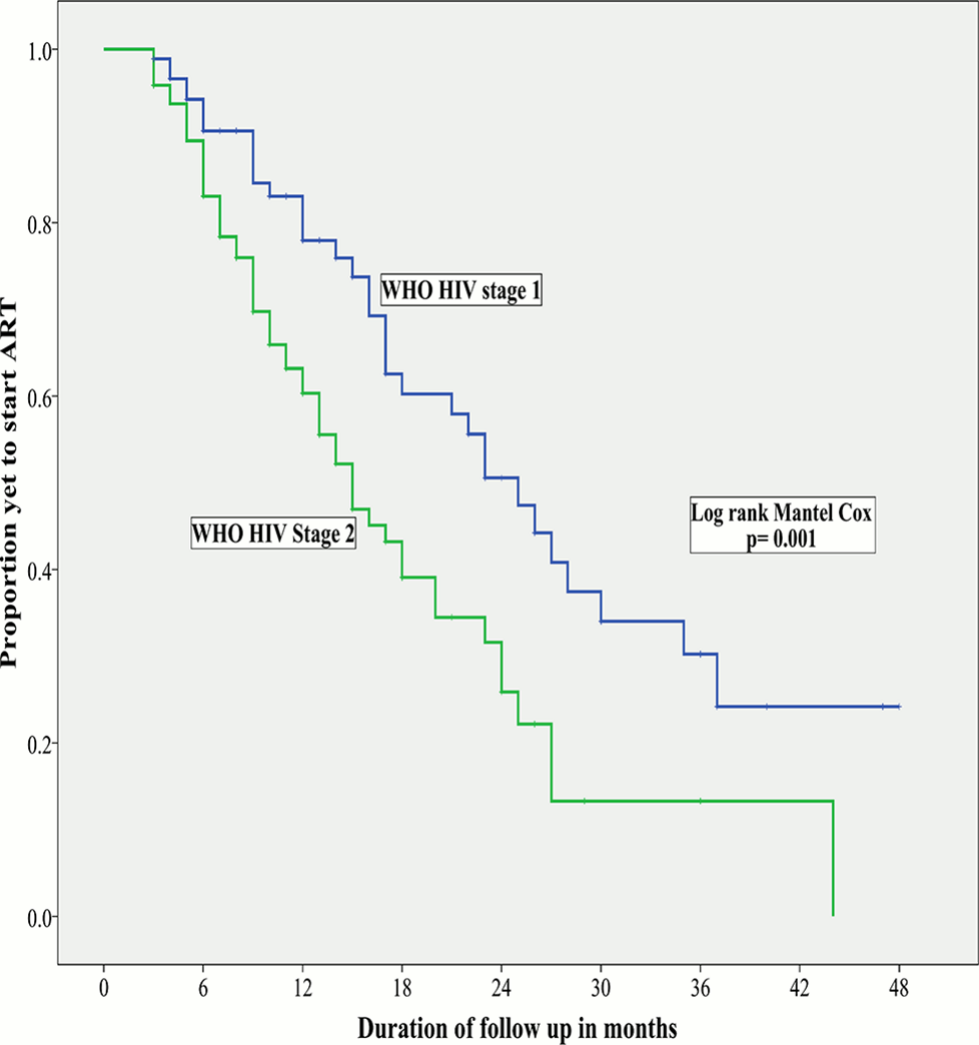
Kaplan-Meier curves of time to initiation of ART in relation to WHO HIV stage. The median time to ART initiation was significantly shorter in participants with stage 2 disease (15months) than participants with stage 1 disease (23months).

The Kaplan-Meier curves in relation to CD4+ cell count ranges and WHO HIV staging are shown in Figs 1 and 2 respectively. The median time to initiation of ART was shorter in study participants with stage 2 disease (15months CI; 11.9–18.1) than those with stage 1 disease (23months, CI; 17.8–28.2, p = 0.001). The median time to initiation of ART was also significantly shorter in study participants with CD4+ cell count of 351-500cells/ul (16months, CI: 10.8–21.2) than those with CD4+ cell count > 500cells/ul (23months, CI: 17.7–28.3, p = 0.002).

### Six-monthly Changes in CD4+ in Relation to ART status

**Fig 3:**
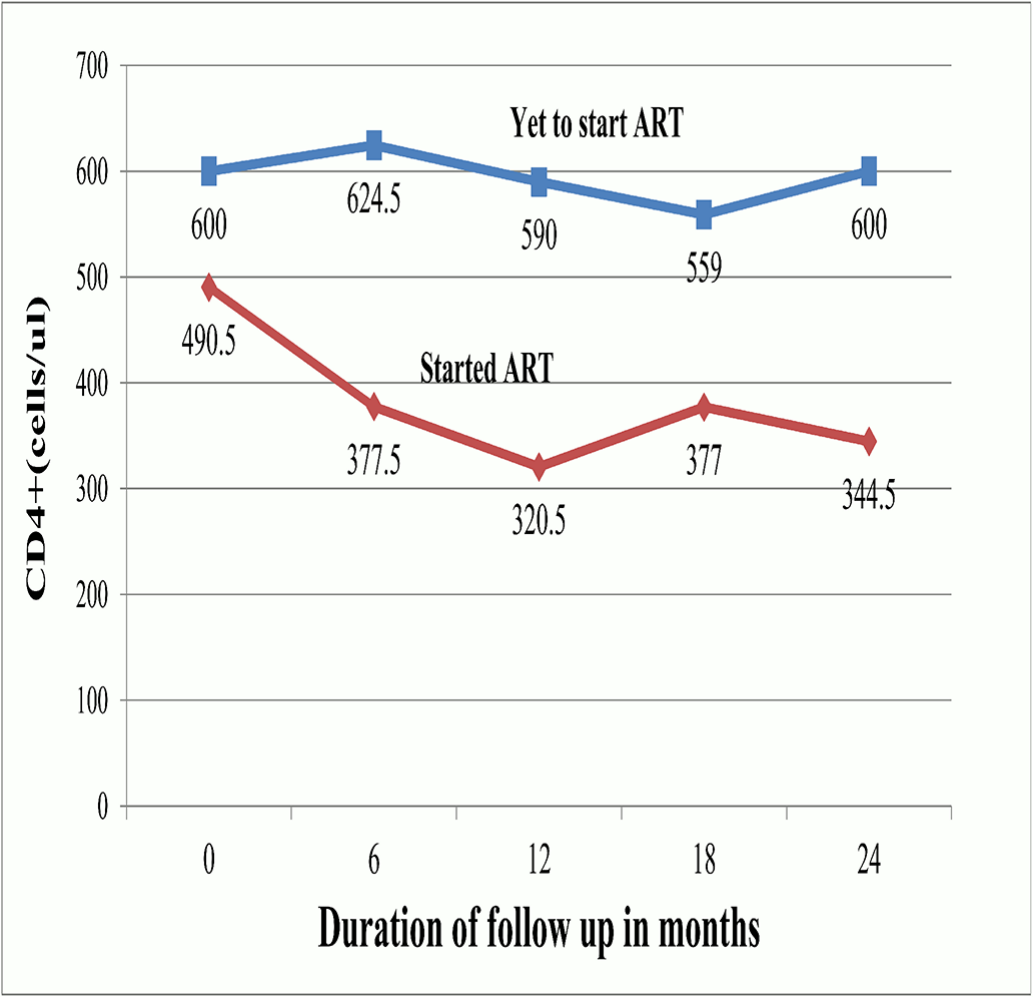
Temporal 6monthly median CD4 cell counts according to ART status. At each individual time points, the observed median CD4 cell counts were significantly higher in participants who were yet to start ART (p<0.0001 at each indivudal time points of 0, 6, 12 and 24 months and p = 0.0001 at 18months; Mann Whitnet test). Among study participants who started ART, the graph shows a steep and progressive drop in median CD4 cell counts in the first year of follow up.

The median CD4+ cell counts obtained at 6 month intervals were significantly lower in participants starting ART as compared to those who did not (Fig 3). Among study participants who started ART, the Fig 3 shows a steep and progressive drop in median CD4 cell counts in the first year of follow up.

## Discusssion

In this study conducted in a region with generalised HIV epidemic in Nigeria, our results have shown that the WHO HIV staging and CD4+ are both independent predictors of timing of ART initiation among adults ineligible for ART. Study participants who at baseline had stage 2 disease or CD4+ of 351-500cells/ul had approximately two times risk of initiating ART earlier than those with stage 1 disease or CD4+ of >500cells/ul. Furthermore, study participants with baseline CD4+ of 351-500cells/ul were about two times more likely to have symptomatic stage 3 disease such as recurrent diarrhoea and pulmonary tuberculosis than those with baseline CD4+ >500cells/ul. We also observed steep CD4+deterioration, especially in the first year of follow up, among participants who subsequently started ART as a compared to those who did not.

Overall, our results corroborate findings from various other observational and randomised prospective studies[4][11][12][2], including mathematical models[7], mainly from the developed world, which have shown that ART-naïve individuals with CD4+ count of 351 to 500cells/ul develop faster clinical and immunological deterioration, as well as higher mortality than ART-naïve individuals with CD4+ count of >500cells/ul. The clinical spectrum of presentation in our cohort is similar to another cohort from Cote d’ Ivoire where pulmonary tuberculosis, recurrent diarrhoea and bacterial infections were reported as common clinical events on follow up of untreated adults before initiation of ART[13].

We observed a high LTFU rate of 34% among our study participants. Poor retention in HIV care remains a challenge in Africa[14], Nigeria inclusive[15][16][17][18], with only about 45% pre-ART clients retained in care in Sub-Saharan Africa[19]. It is also probable that these clients have informally transfered to other ART treatment sites or perhaps died at home [20]. The high rates of LTFU has implications for HIV transmission as these individuals serve as reservoirs for HIV-transmission to uninfected population if they engage in risky behaviours. Furthermore, these HIV-infected individuals may inadvertently present late for care at a time when they have become symptomatic or developed AIDS. Strengthening of post-test counselling services, regular visits for cotrimoxazole prophylaxis, decentralization of care, and earlier initation of ART, among others, may improve HIV-infected patient retention rates[19].

Since commencement of ART is beneficial in preventing progression of HIV disease, our results provide evidence to recommend earlier initiating of ART in Nigeria among all symptomatic HIV-infected individuals with stage 2 disease, irrespective of the CD4+ count and among individuals with CD4+ count of 500cells or less, irrespective of the WHO HIV stage. It may also be necessary to consider earlier initiation of ART in individuals with steep fall in serial CD4+ count especially in the first year of follow up. However, this recommendation is supported only by a few studies[21][22], with randomized studies suggesting no added value of using rate of fall in CD4 cell count in deciding when to start ART.

Although our study is not prospectively designed, our results also seem to suggest that earlier initiation of ART in individuals with CD4 cell count of 351-500cells/ul may be possibly beneficial in preventing development of tuberculosis, diarrhoea and weight loss. The benefit of ART initiation in preventing tuberculosis is supported by various other studies from around the world[23][24].

Despite the clinical benefit of earlier initiation of ART in certain groups of HIV-infected individuals, successful implementation of this guideline remains a major constraint in Nigeria due to funding gaps, poor infrastructure, weak health systems and shortage of health manpower, among other challenges [9] [25]. Furthermore, the benefits and cost effectiveness of earlier initiation of ART are dependent on a high testing uptake, high treatment coverage, sustained adherence and high rates of retention in care[1]. Unfortunately, all the foregoing remain challenges of HIV care in Nigeria deserving priority attention[9] [26][18][25].

The major limitation to our study is the retrospective study design which meant we had to contend with some missing data. However, it is our view that the major conclusions of our study were not significantly affected by missing data. Future prospective studies on natural history of HIV-infection among adults in Nigeria are desirable. Such studies should also ascertain the influence of other possible determinants of HIV progression such as HIV viral load, and Hepatitis B and C infections, among others[27], which we could not assess in our study due to resource constraints. The high rates of LTFU in our study probably reduced the statistical power of our results. However, we believe that this limitation did not significantly influence the determination of predictors of timing of ART initiation since we restricted our analysis to study participants followed up for at least 3months while majority of LTFU occurred by one month of follow up.

In conclusion, our results have shown that it may be clinically beneficial to initiate ART earlier in HIV-infected adults from Nigeria with WHO HIV stage 2 and or CD4+ of 351-500cells/ul. Overall, our findings support the current WHO recommendation for upward review of time to start ART from CD4 cell count of 350cells/ul to <500cells/ul

## Supporting Information

S1 Dataset Data sheet of all 280 study participants. (SAV)

S2 Dataset Data sheet of all study participants followed up for at least 3months. (SAV)

S1 Results Results of associations between lost to follow up and study variables. (PDF)
