# Supplementary material for: Factors Associated with Timing of Initiation of Antiretroviral Therapy among HIV-1 Infected Adults in the Niger Delta Region of Nigeria
Source: PLoS One. 2015 May 1;10(5):e0125665. doi: 10.1371/journal.pone.0125665 (PMC4416715; doi:10.1371/journal.pone.0125665)
Supplement: S1 Results — (PDF) [file pone.0125665.s003.pdf]

Means

[DataSet2] C:\Users\AllennellaLensMedia\Documents\Timing of ART\TIME TO IN IATION OF AR Nigeria.sav

Case Processing Summary

	Cases					
	Included		Excluded		Total	
	N	Percent	N	Percent	N	Percent
AGE * Lost to follow up group	280	100.0%	0	0.0%	280	100.0%
Baseline CD4 cell count * Lost to follow up group	280	100.0%	0	0.0%	280	100.0%
Baseline packed cell volume * Lost to follow up group	219	78.2%	61	21.8%	280	100.0%
Baseline Creatinine clearance new * Lost to follow up group	183	65.4%	97	34.6%	280	100.0%
Fasting blood sugar * Lost to follow up group	172	61.4%	108	38.6%	280	100.0%

Report

Lost to follow up group		AGE	Baseline CD4 cell count	Baseline packed cell volume	Baseline Creatinine clearance new
LTFU	Mean	33.16	622.8542	34.6522	88.7698
	N	96	96	67	56
	Std. Deviation	9.996	206.47814	5.25796	28.24422
Not LTFU	Mean	32.72	587.9457	35.0908	89.4979
	N	184	184	152	127
	Std. Deviation	9.127	183.16068	4.71777	30.13727
Total	Mean	32.87	599.9143	34.9566	89.2751
	N	280	280	219	183
	Std. Deviation	9.418	191.82472	4.88137	29.49541

Report

Lost to follow up group		Fasting blood sugar
LTFU	Mean	5.0463
	N	54
	Std. Deviation	1.75872
Not LTFU	Mean	4.9096
	N	118
	Std. Deviation	1.44139
Total	Mean	4.9525
	N	172
	Std. Deviation	1.54410

ANOVA Table

			Sum of Squares	df
AGE * Lost to follow up group	Between Groups	(Combined)	12.150	1
	Within Groups		24735.961	278
	Total		24748.111	279
Baseline CD4 cell count * Lost to follow up group	Between Groups	(Combined)	76876.528	1
	Within Groups		10189409.41	278
	Total		10266285.94	279
Baseline packed cell volume * Lost to follow up group	Between Groups	(Combined)	8.944	1
	Within Groups		5185.514	217
	Total		5194.458	218
Baseline Creatinine clearance new * Lost to follow up group	Between Groups	(Combined)	20.602	1
	Within Groups		158315.612	181
	Total		158336.215	182
Fasting blood sugar * Lost to follow up group	Between Groups	(Combined)	.692	1
	Within Groups		407.014	170
	Total		407.707	171

ANOVA Table

			Mean Square	F	Sig.
AGE * Lost to follow up group	Between Groups	(Combined)	12.150	.137	.712
	Within Groups		88.978		
	Total				
Baseline CD4 cell count * Lost to follow up group	Between Groups	(Combined)	76876.528	2.097	.149
	Within Groups		36652.552		
	Total				
Baseline packed cell volume * Lost to follow up group	Between Groups	(Combined)	8.944	.374	.541
	Within Groups		23.896		
	Total				
Baseline Creatinine clearance new * Lost to follow up group	Between Groups	(Combined)	20.602	.024	.878
	Within Groups		874.672		
	Total				
Fasting blood sugar * Lost to follow up group	Between Groups	(Combined)	.692	.289	.591
	Within Groups		2.394		
	Total				

Relationship between study variables and lost to follow up status

		Lost to follow up group		
		LTFU		Not LTFU
		Count	Row Valid N %	Count
SEX	MALE	32	38.6%	51
	FEMALE	64	32.5%	133
MARITAL STATUS	EVER MARRIED	48	29.8%	113
	NEVER MARRIED	38	39.2%	59
HIV stage at presentation	STAGE 1	45	33.1%	91
	STAGE 2	50	35.0%	93
Baseline CD4 GROUP	350-500	30	28.6%	75
	>500	66	37.7%	109
Baseline CREATININE CLEARANCE group	>90MLS/MIN	25	33.3%	50
	<90MLS/MIN	31	28.7%	77

Relationship between study variables and lost to follow up status

		Lost to follow up group		
		Not LTFU	Total	
		Row Valid N %	Count	Row Valid N %
SEX	MALE	61.4%	83	100.0%
	FEMALE	67.5%	197	100.0%
MARITAL STATUS	EVER MARRIED	70.2%	161	100.0%
	NEVER MARRIED	60.8%	97	100.0%
HIV stage at presentation	STAGE 1	66.9%	136	100.0%
	STAGE 2	65.0%	143	100.0%
Baseline CD4 GROUP	350-500	71.4%	105	100.0%
	>500	62.3%	175	100.0%
Baseline CREATININE CLEARANCE group	>90MLS/MIN	66.7%	75	100.0%
	<90MLS/MIN	71.3%	108	100.0%

Pearson Chi-Square Tests

		Lost to follow up group
SEX	Chi-square	.954
	df	1
	Sig.	.329
MARITAL STATUS	Chi-square	2.387
	df	1
	Sig.	.122
HIV stage at presentation	Chi-square	.109
	df	1
	Sig.	.741
Baseline CD4 GROUP	Chi-square	2.435
	df	1
	Sig.	.119
Baseline CREATININE CLEARANCE group	Chi-square	.447
	df	1
	Sig.	.504

Results are based on nonempty rows and columns in each innermost subtable.
